# Ciclosonide reduces lipid droplet burden in APOE4 astrocytes

**DOI:** 10.1002/alz70859_102039

**Published:** 2025-12-25

**Authors:** Jasmine Phenix, Sarah Anna Lisa Zünd, Dongwei Yu, Antonio Vázquez Cobá, Marina Ruelas Hernandez, Nicolas Audet, Lisa Marie Munter

**Affiliations:** ^1^ McGill University, Montreal, QC Canada; ^2^ Technische Universität München, Munich, Bavaria Germany; ^3^ McGill University, Montréal, QC Canada

## Abstract

**Background:**

Prior studies have shown that ApoE ε4 astrocytes display a higher LD count than ApoE ε3 astrocytes. We examined the effects of pharmacological agents on lipid droplets (LDs) in apolipoprotein E (ApoE) ε3 and ApoE ε4 astrocytes.

**Method:**

We hypothesized that drugs reducing LDs in ApoE ε4 astrocytes could reveal mechanisms of LD formation and inform potential treatments for AD. A screen of 2,321 FDA‐approved drugs was conducted to identify candidates for investigating LD differences between ApoE ε3 and ApoE ε4 astrocytes.

**Result:**

Both ApoE ε3 and ApoE ε4 astrocytes produced LDs detectable with BODIPY staining. PLIN2 co‐staining ensured LD specificity. A drug screen and validation workflow identified three compounds, ciclosonide, VX745, and ipragliflozine, as reducing LDs in ApoE ε4 astrocytes specifically. Ciclesonide, a glucocorticoid with a known mechanism of action, showed dose‐dependent effects on LD reduction (0.05, 0.5, and 5 mM). Transcriptomic analysis of ciclesonide‐treated astrocytes revealed significant changes in extracellular matrix organization.

**Conclusion:**

These findings enhance understanding of LD formation and isoform‐specific differences in astrocytes. Given the role of LDs in AD, these insights may contribute to understanding AD pathogenesis and identifying therapeutic strategies.

